# Supplementary material for: Metabolomic Analysis on the Mechanism of Nanoselenium Biofortification Improving the Siraitia grosvenorii Nutritional and Health Value
Source: Foods. 2022 Sep 29;11(19):3019. doi: 10.3390/foods11193019 (PMC9564208; doi:10.3390/foods11193019)
Supplement: Supplementary file 1 [file foods-11-03019-s001.zip › supplementary Figures and Tables S1-S7.pdf]

Metabolomic analysis on the mechanism of nanoselenium biofortification improving the *Siraitia grosvenorii* nutritional and health value

Chunran Zhou [1], Jingbang Zhang [1], Yangliu Wu [1], Haiyan Cheng [1], Qiuling Pang [2], Yuanhui Xiao [2], Dong Li [3,*]
and Canping Pan [1,*]

[1] Innovation Center of Pesticide Research, Department of Applied Chemistry, College of Science, China Agricultural University, China Yuanmingyuan West Road 2, Beijing 100193, China
[2] Guangxi Academy of Specialty Crops, Putuo Road 40, Guilin 541004, China
[3] Key Laboratory of Green Prevention and Control of Tropical Plant Diseases and Pests, Ministry of Education, College of Plant Protection, Hainan University, Haikou 570228, China
* Correspondence: lidong1105@hainanu.edu.cn (D.L.); canpingp@cau.edu.cn (C.P.)

Total number of pages: 11

Total number of figures: 2

Total number of tables: 7

SUPPORTING TABLES AND FIGURES

Figure S1. Effect of nano-Se on Se contents in *Siraitia grosvenorii*

Figure S2. Effect of nano-Se on the weight of *Siraitia grosvenorii*

Table S1. Parameters for mogroside V analysis by UPLC-MS/MS

Table S2. Parameters for flavone compounds analysis by UPLC-MS/MS

Table S3. Parameters for phenolic acids compounds analysis by UPLC-MS/MS

Table S4. Parameters for carbohydrate compounds analysis by ICS-5000^{+}

Table S5. Parameters for amino acids compounds analysis by UPLC-HRMS

Table S6. Qualitative compounds listed by GC-IMS analysis

Table S7. The contents of volatile compounds treated by control and nano-Se

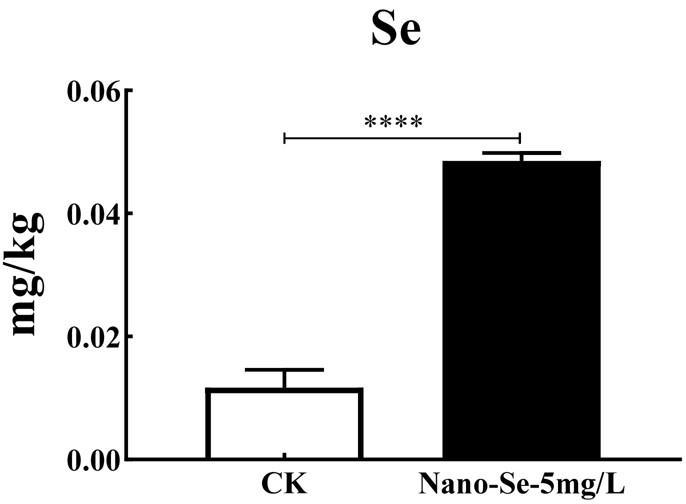

Figure S1. Effect of nano-Se on Se contents in *Siraitia grosvenorii*. Data are presented as mean ± SEM (**** $P < 0.0001$. n = 3).

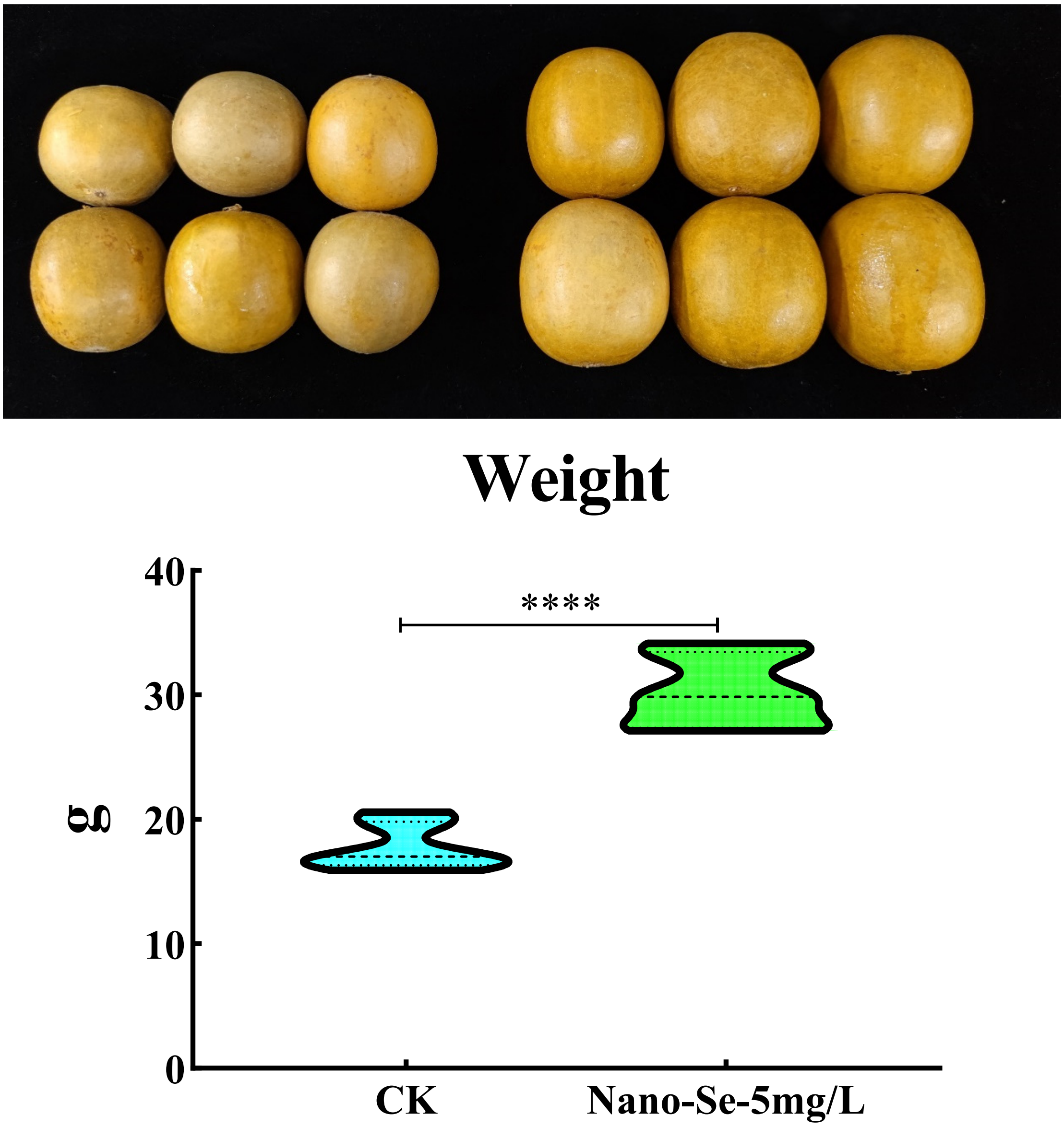

Figure S2. Effect of nano-Se on the weight of *Siraitia grosvenorii*. Data are presented as mean ± SEM (**** $P < 0.0001$. n = 6).

Table S1. Parameters for mogroside V analysis by UPLC-MS/MS

Name	Precursor/quantitative transition(m/z)	Precursor/qualitative transition(m/z)	Retention time(min)	Collision energy (CE)(V)	Polarity	Delta EMV
mogroside V	1309.5/1309.5	1309.5/1309.5	0.47	120(5)	Positive	500

Table S2. Parameters for flavone compounds analysis by LC-MS/MS

Name	Precursor/quantitative transition (m/z)	Precursor/qualitative transition (m/z)	Retention time(min)	Collision energy (CE)(V)	Polarity	Delta EMV (-)
Apigenin	269/117	269/225	2.982	150(40)	Negative	300
Luteolin	285/133	285/175	1.956	160(35)	Negative	300
Quercetin	301/151	301/178.9	2.068	140(20)	Negative	300
Kaempferol	285/117	285/153	3.354	155(45)	Negative	300

Table S3. Parameters for phenolic acids compounds analysis by LC-MS/MS

Name	Precursor/quantitative transition(m/z)	Precursor/qualitative transition (m/z)	Retention time(min)	Collision energy (CE)(V)	Polarity	Delta EMV (-)
Ferulic Acid	193/134	193/177.8	2.637	90(18)	Negative	300
Chlorogenic acid	353.1/191.1	353.1/127.1	3.37	65(15)	Negative	300
Caffeic acid	179/135	179/79.1	0.964	160(15)	Negative	300
4-Hydroxy-3,5-dimethoxycinnamic acid	223.1/208	223.1/163.8	7.718	90(10)	Negative	300
4-Hydroxybenzoic acid	137/93.1	137/65.1	8.592	70(15)	Negative	300
Syringic acid	197/182	197/167	4.279	70(10)	Negative	300

Table S4. Parameters for carbohydrate compounds analysis by ICS-5000^{+}

gradient elution program

Time (min)	Flow (mL/min)	%A	%D
0	1	91	9
18	1	91	9
21	1	0	100
31	1	0	100
32	1	91	9
40	1	91	9

column cleaning procedure

Time (min)	Flow (mL/min)	%A	%C	%D
0	1	91	0	9
4	1	91	0	9
6	1	0	50	50
30	1	0	50	50
31	1	91	0	9
40	1	91	0	9

Table S5. Parameters for amino acids compounds analysis by UPLC-HRMS

Time (min)	Flow (mL/min)	%A	%B
0	0.3	97	3
2	0.3	88	12
9.5	0.3	76	24
10	0.3	0	100
12	0.3	0	100
12.1	0.3	97	3
15.5	0.3	97	3

Table S6. Qualitative compounds listed by GC-IMS analysis

Count	Compound	CAS#	Formula	MW	RI	Rt [sec]	Dt [RIPrel]
1	Butyl acetate monomer	123-86-4	$C_6H_{12}O_2$	116.2	803.1	273.777	1.23792
2	Butyl acetate dimer	123-86-4	$C_6H_{12}O_2$	116.2	801.4	271.962	1.61759
3	2-Heptanone monomer	110-43-0	$C_7H_{14}O$	114.2	887.9	367.227	1.25989
4	2-Heptanone dimer	110-43-0	$C_7H_{14}O$	114.2	888.3	367.681	1.62857
5	hexanal monomer	66-25-1	$C_6H_{12}O$	100.2	787.5	256.596	1.25178
6	hexanal dimer	66-25-1	$C_6H_{12}O$	100.2	787.3	256.4	1.56079
7	n-Nonanal monomer	124-19-6	$C_9H_{18}O$	142.2	1104.1	766.924	1.47358
8	n-Nonanal dimer	124-19-6	$C_9H_{18}O$	142.2	1103.6	765.852	1.94422
9	ethyl hexanoate monomer	123-66-0	$C_8H_{16}O_2$	144.2	996.9	557.891	1.33912
10	ethyl hexanoate dimer	123-66-0	$C_8H_{16}O_2$	144.2	1000.7	565.395	1.81424
11	(E)-2-octenal monomer	2548-87-0	$C_8H_{14}O$	126.2	1053	667.231	1.32567
12	(E)-2-octenal dimer	2548-87-0	$C_8H_{14}O$	126.2	1053	667.231	1.81872
13	Heptanal monomer	111-71-7	$C_7H_{14}O$	114.2	910.7	404.782	1.34572
14	Heptanal dimer	111-71-7	$C_7H_{14}O$	114.2	906.6	397.485	1.69908
15	1-Pentanol monomer	71-41-0	$C_5H_{12}O$	88.1	767.2	239.402	1.25356
16	1-Pentanol dimer	71-41-0	$C_5H_{12}O$	88.1	764.4	237.217	1.50799
17	3-methylbutanal	590-86-3	$C_5H_{10}O$	86.1	634.6	153.135	1.40481
18	2-Pentanone	107-87-9	$C_5H_{10}O$	86.1	678.6	172.319	1.37684
19	Pentanal	110-62-3	$C_5H_{10}O$	86.1	694.8	181.677	1.41996
20	Acetone	67-64-1	C_3H_6O	58.1	522.4	104.106	1.12135
21	ethanol	64-17-5	C_2H_6O	46.1	484.8	87.687	1.04015
22	Propanal	123-38-6	C_3H_6O	58.1	500.9	94.757	1.05387
23	acetic acid	64-19-7	$C_2H_4O_2$	60.1	599.8	137.909	1.15202
24	Methylpropanal	78-84-2	C_4H_8O	72.1	565.7	123.037	1.28289
25	2-Butanone	78-93-3	C_4H_8O	72.1	579.1	128.889	1.24912
26	ethyl acetate	141-78-6	$C_4H_8O_2$	88.1	600.9	138.397	1.33249
27	2-heptenal (E) monomer	18829-55-5	$C_7H_{12}O$	112.2	956.1	485.394	1.25724
28	2-heptenal (E) dimer	18829-55-5	$C_7H_{12}O$	112.2	954.2	482.118	1.66727
29	benzaldehyde	100-52-7	C_7H_6O	106.1	952.4	478.842	1.47828
30	Octanal	124-13-0	$C_8H_{16}O$	128.2	1007	577.668	1.4091

Table S7. The contents of volatile compounds treated by control and nano-Se (**** $P < 0.0001$. n=3).

Compound	Peak area						Peak area						Type	Significance
	CK-1	CK-2	CK-3	CK-4	CK-5	CK-6	Nano-Se-1	Nano-Se-2	Nano-Se-3	Nano-Se-4	Nano-Se-5	Nano-Se-6		
1-Pentanol dimer	287.43	282.34	300.91	305.40	296.25	305.43	440.50	443.00	455.02	469.60	457.11	451.80	up	****
2-Butanone	1033.13	973.36	979.86	997.80	980.65	918.94	1466.26	1482.98	1537.21	1476.20	1496.07	1480.20	up	****
2-Pentanone	3625.08	3815.92	3784.75	3806.79	3706.56	3658.02	5382.75	5353.55	5441.99	5485.59	5503.06	5517.73	up	****
3-methylbutanal	5659.50	5443.11	5421.97	5487.77	5503.70	5482.16	6551.72	6474.83	6462.59	6485.78	6532.95	6542.70	up	****
ethanol	965.92	951.54	952.95	941.77	996.86	927.83	1428.04	1403.04	1358.20	1306.91	1433.27	1341.24	up	****
Heptanal dimer	9403.19	9346.44	9524.52	9444.45	9570.81	9310.74	10646.30	10677.16	10804.33	10501.57	10560.82	10571.14	up	****
Methylpropanal	1243.21	1229.53	1189.87	1189.81	1230.05	1159.41	2893.53	2898.17	2944.68	3019.13	3044.13	3037.38	up	****
n-Nonanal dimer	2114.77	2204.67	2181.41	2190.37	2188.64	2024.77	5194.88	5199.08	5219.93	5241.20	5170.17	5168.09	up	****
Octanal	1069.63	1043.28	1065.09	1087.55	1052.34	1067.25	1503.40	1527.34	1579.70	1546.83	1539.29	1520.81	up	****
Pentanal	2156.09	2163.55	2167.28	2176.74	2160.34	2147.38	3348.41	3350.59	3348.37	3230.90	3246.67	3254.14	up	****
Acetone	3810.33	3850.89	3820.48	3846.24	3654.26	3616.00	9755.51	9704.15	9856.98	9932.99	9991.17	10050.31	up	****
(E)-2-octenal dimer	26952.53	27057.00	27044.87	26683.84	27002.59	26688.85	20738.41	20786.06	20730.62	20369.05	20537.10	20501.46	down	****
2-heptenal (E) dimer	2045.61	1866.18	1780.41	1870.02	2048.87	1749.06	758.14	776.00	768.85	655.32	614.38	610.48	down	****
benzaldehyde	1054.30	1153.71	1174.55	1248.32	1377.14	1200.87	485.98	517.62	504.31	468.42	478.91	440.43	down	****
ethyl acetate	656.53	654.24	640.60	632.80	626.12	696.80	509.28	521.63	551.07	544.76	544.57	531.86	down	****
Propanal	1862.45	1789.32	1692.22	1650.69	1631.88	1615.82	967.53	999.19	967.58	935.72	937.39	905.50	down	****
